# Deep learning approach for crop-weed segmentation in peanut cultivation using PSPEdgeWeedNet

**DOI:** 10.1038/s41598-025-24174-6

**Published:** 2025-12-03

**Authors:** Deepthi G Pai, Mamatha Balachandra, Radhika Kamath

**Affiliations:** Manipal Institute of Technology, Manipal Academy of Higher Education, Manipal, Karnataka 576104 India

**Keywords:** Boundary loss, Crop, Edge aware networks, Multiclass segmentation, Object detection, Precision agriculture, PSPNet, Segmentation, Weed, Plant sciences, Environmental sciences, Engineering

## Abstract

Weed management continues to be a significant challenge in modern agriculture, primarily due to the aggressive growth patterns of weeds and their direct competition with crops for essential resources such as light, water, and nutrients. Although recent developments in precision agriculture have led to the emergence of automated weed detection systems aimed at reducing operational costs and decreasing reliance on chemical herbicides, achieving accurate crop–weed segmentation remains a persistent difficulty. This is largely attributed to high visual similarity between crops and weeds, coupled with variations in illumination and field conditions. To address these challenges, Convolutional Neural Networks (CNNs) have been increasingly adopted for their capability to perform end-to-end, pixel-level classification, particularly when leveraging multi-spectral imagery. In this context, PSPEdgeWeedNet is proposed, a novel edge-aware deep learning architecture tailored for precise semantic segmentation of crops and weeds within peanut cultivation fields. Distinct from the conventional Pyramid Scene Parsing Network (PSPNet) and its boundary-aware variant developed as a baseline in this research, PSPEdgeWeedNet introduces a dedicated edge detection branch. This branch is specifically engineered to enhance boundary localization and improve delineation between adjacent vegetation classes. In post-processing, Conditional Random Fields (CRFs) are used to slightly enhance the segmentation results around object boundaries. Additionally, all models were trained on a curated peanut field dataset using class-weighted loss functions to effectively address inherent class imbalance. Comprehensive experimental evaluations reveal that PSPEdgeWeedNet significantly outperforms existing state-of-the-art architectures including PSPNet, SegNet, UNet, DeepLabv3, Swin-Unet, and light weight transformer model based on ViT across multiple performance metrics such as Intersection over Union (IoU), precision, recall, and F1-score. These results highlight the critical role of incorporating edge-aware mechanisms within semantic segmentation frameworks, thereby enhancing the robustness and accuracy of automated weed detection systems in complex, real-world agricultural environments.

## Introduction

Weeds, often referred to as “plants out of place,” compete with crops for essential resources such as soil nutrients and water, leading to distinctive crop-weed interactions that can significantly reduce crop yields^1^. However, the importance of weed management is often underestimated by both farmers and the general public due to the high costs associated with herbicides and manual weeding. Additionally, weeds serve as hosts for pests that can damage crops, further exacerbating agricultural losses^2^. These weeds can also hinder crop growth. Therefore, it is essential to accurately identify and detect them^3^.

Arachis hypogaea L., or peanuts(groundnuts), are a great source of important nutrients and health advantages. However, several factors, including disease, insects, drought, climate change, soil fertility, and weed infestation, have an impact on peanut crop yields. Peanut production losses of up to 70% occur during the crucial 40–45 day post-sowing phase for crop-weed competition^4^. Therefore, detecting and removing weeds from peanut farms is essential to enhance crop yield.

**Fig. 1:**
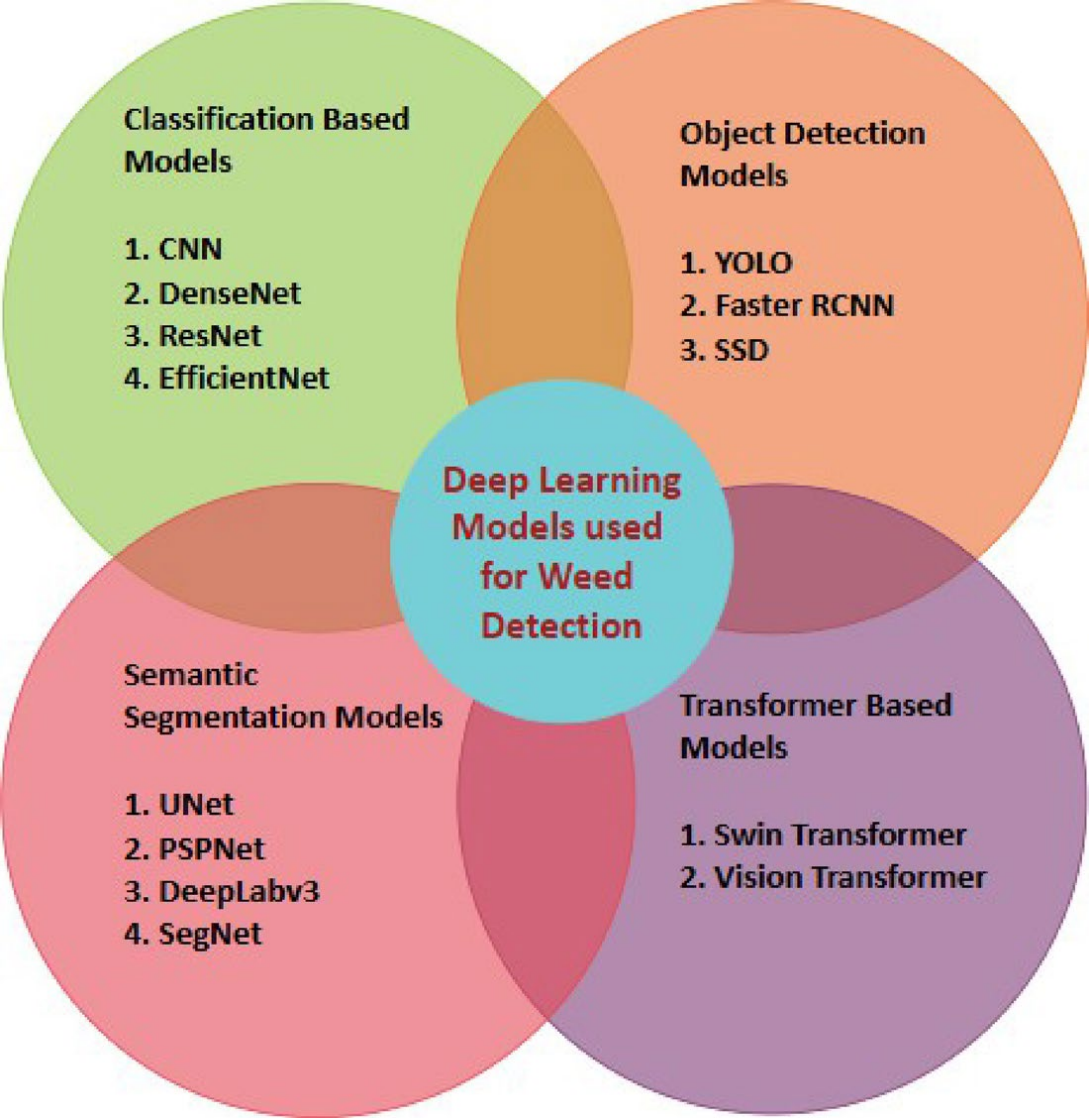
Deep Learning models used in weed detection.

Artificial Intelligence (AI) can play a significant role in agricultural tasks, with Deep Learning models enabling accurate classification and detection of weeds and crops in the field^5,6^. Crop monitoring, yield forecasting, disease detection, soil health management, climate forecasting, supply chain optimization, genomics and breeding, precision agriculture, and market analysis have all been made possible by Deep Learning (DL), which has completely changed the agricultural industry^7^. These technologies analyze data from satellites, drones, and Internet of Things devices to identify weeds and pests, monitor crop health, diagnose crop and leaf diseases, estimate yields, and optimize fertilization and irrigation^8^. Additionally, they aid in the early identification of crop diseases, enabling timely action to prevent crop loss. DL models support breeding and genomics, supply chain optimization, and climate predictions^9^. DL-based precision agriculture uses real-time data to optimize resource utilization and reduce environmental impacts^10–12^. Figure 1 shows different DL models used for weed detection purposes.

Semantic segmentation, a key technique in DL, can be effectively utilized for crop and weed detection purposes^13–15^. Every pixel in the image is classified into one of the pre existing classes using semantic segmentation. As a result, it offers more accurate crop and weed location data^16^. These Deep Neural Net based techniques, which have many tuning parameters, usually need a few thousand to tens of thousands of images to train to provide sufficient performance and prevent overfitting^16^. However, standard segmentation models often struggle with accurately distinguishing weed boundaries, leading to misclassification errors^17^.

One of the widely used models for semantic segmentation is PSPNet (Pyramid Scene Parsing Network)^18^. However, it has certain limitations, including poor boundary delineation, low Intersection over Union (IoU) for small and rare classes, and high sensitivity to background noise. Despite the growing use of DL models in precision agriculture, accurately segmenting crops and weeds in field conditions remains a challenging task due to high visual similarity between classes, varying illumination, and complex backgrounds. Existing semantic segmentation models such as UNet, SegNet, DeepLabV3, PSPNet, and transformer- based architectures like Swin-UNet and lightweight Vision Transformer (ViT) variants often struggle with precise boundary delineation, leading to misclassifications, particularly in tightly clustered or overlapping vegetation.

To address this gap, the authors propose PSPEdgeWeedNet, a novel semantic segmentation architecture designed specifically for crop–weed discrimination in peanut fields. The model integrates a boundary-aware loss function and a dedicated edge detection branch to enhance localization at object borders. Furthermore, Conditional Random Fields (CRFs) are used in post-processing to refine the segmentation masks. The model was also qualitatively evaluated on a separate peanut dataset collected from the Udupi region of Karnataka, which contains different weed species and varying lighting conditions. While the model was not trained on this dataset, it successfully identified every crop instance, indicating promising generalization capabilities.Experimental results demonstrate that PSPEdgeWeedNet outperforms the aforementioned models across all major performance metrics, including precision, recall, F1-score, and Intersection over Union (IoU), thereby establishing its effectiveness in handling the nuanced challenges of field-based weed detection.

The model is thoroughly tested on a peanut crop-weed dataset and compared against the following methods:

- PSPNet, serving as the baseline semantic segmentation model.
- Boundary-aware PSPNet, which integrates a boundary-sensitive loss function and investigates different class weighting schemes to address class imbalance.
- Other widely used semantic segmentation models, including UNet, SegNet, and DeepLabv3, Swin-Unet, light weight transformer model based on ViT.
- The model was tested on a peanut dataset collected from the Udupi region, which includes diverse weed species and varying lighting conditions.

This study aims to develop a novel PSPNet-based architecture augmented with a boundary-aware loss function and an edge detection branch, aiming to improve boundary accuracy while maintaining computational efficiency suitable for real-time deployment.

### The significance of edge detection in segmentation models

The primary goal of edge detection is to pinpoint the specific regions or pixels within an image that represent boundaries or transitions—typically where there are sharp changes in intensity or color—indicating the presence of edges^19^. The technique’s working premise states that an edge is any pixel in the chosen band of the RGB image that has a value different from that of its neighbors^20^.

Edge detection is a crucial technique in segmentation models, particularly in tasks like crop and weed detection^21^. It enhances object boundaries, reduces misclassification, differentiates objects with similar textures or colors, and prevents background noise interference. Edge detection also improves IoU for small objects, preserving their structure and boosting segmentation performance. It complements global context learning by adding local fine-grained details, leading to more precise segmentation masks. In this study, edge detection refers to the limits of crop, weed, and background, indicating the significant difference between the object and nearby objects. Sobel edge detection is a gradient-based method that determines the rate at which an image’s intensity changes and uses the gradient magnitude to identify both horizontal and vertical edges^22^. The Sobel filter in the early layers of the proposed PSPEdgeWeedNet model improves segmentation efficiency and reduces noise, thereby boosting edge detection performance.

## Motivation of this Study

Despite reaching excellent overall accuracy in agricultural applications, current semantic segmentation algorithms often fail to provide the precise border delineation required for successful precision farming. This paper proposes a novel PSPNet-based architecture to bridge the crucial gap in boundary-aware agricultural segmentation, enhanced by boundary-aware loss functions and a dedicated edge detection branch. The proposed approach focuses on the difficulty of accurately identifying crop-weed boundaries, which is important for precision mechanical weeding, focused herbicide application, and autonomous agricultural operations. The suggested framework prioritizes edge-critical areas, as opposed to previous approaches that provide equal weight to every pixel. For real-time agricultural applications, this results in notably improved boundary accuracy while maintaining computing efficiency.

## Related works

With its exceptional ability to differentiate between crops and weeds in a variety of agricultural settings, semantic segmentation has become a game-changing technique in precision agriculture. Several research projects have achieved F1-scores above 90% and mean Intersection over Union (mIoU) values as high as 92%, demonstrating the technology’s consistently outstanding performance metrics. Table 1 summarizes different DL methods utilized in agricultural domains, detailing their aim, model architectures, datasets used, and associated limitations.

**Table 1:** Analysis of different deep learning techniques in the agricultural domain.

Citation	Aim of the paper	Models or algorithms	Dataset	Limitations
^23^	To improve pixel-level precision in distinguishing crop (tobacco) and weed pixels using a two-stage semantic segmentation approach, enhancing classification performance over traditional one- stage models	Stage 1(Binary classifier) Stage 2 (three class clas- sifier)	A newly captured aerial tobacco crop dataset	The two-stage approach doubles the computation compared to one-stage models and even though inference time is still acceptable for real-time, it is costlier computationally
^13^	Weed and crop segmentation network that improves performance in recognizing weeds of any shape in complex environments	DNN	Stuttgart and Bonn datasets	Lack of Generalization Evidence Resource Demanding
^26^	Detects vegetation outside the crop mask as weeds by combining semantic segmentation and image processing	CCNet GCNet ISANet DeepLabV3 DeepLabV3 +	Cornfields near Shenyang, Liaoning Province, China	Limited Temporal and Geographic Diversity
^24^	An automated semantic segmentation- based approach for multiclass weed identification in precision agriculture	U-Net based on Inception-ResNetV2	Brinjal farms in Gorakhpur, Uttar Pradesh, India	Use of RGB Images only Assumption of Uniform Lighting and Weather Conditions
^25^	Advanced semantic segmentation model for accurate crop–weed differentiation, enhancing precision weeding under natural field conditions	EPAnet	Bean sprout cultivation base in Avignon, France	Limited Generalizability
^26^	Lightweight, real-time weed segmentation	CCNet GCNet ISANet DeepLabV3 DeepLabV3 +	Cornfields near Shenyang, Liaoning Province, China	Geographic and Temporal Limitation Indirect Weed Segmentation
^27^	Embeds a SE module for soybean weeds in a genuine field setting and suggests an enhanced UNet structure	UNet ResNet34	Soybean experimental field of Jilin Agricultural University	Real-time deployment on edge devices for in-field precision spraying remains a challenge due to computational complexity
^28^	Weed recognition model based on improved Swin-Unet	UNet Swin-Unet DeepLabv3 Mask R-CNN	Maize test field in Zibo, Shan-dong Province, China	Approach may be less effective in scenarios where crops and weeds are heavily interwoven or where morphological differences are subtle, leading to potential misclassification
^29^	Suggest an enhanced model based on YOLOv4 for detecting weeds in potato fields	Improved YOLOv4 al-gorithm	Images were taken from the test site in China	Data set collected from an experimental field, not from the real field
^30^	Proposes a Faster R-CNN network architecture for identifying weeds in cropping region images	Faster RCNN FPN Improved ResNeXt101	V2 Plant Seedlings Dataset	The paper lacks details on the potential effects of lighting conditions or image quality on the model’s performance

### Advanced neural network architectures and performance achievements

Agricultural segmentation challenges have demonstrated the effectiveness of DL systems. On brinjal (eggplant) farm datasets, a U-Net model with Inception-ResNetV2 backbone architecture obtained an F1-score of 96.78%, proving the value of fusing robust feature extraction networks with segmentation frameworks^24^. Researchers created an ERFNet-based multi-decoder architecture with a dual-loss function mechanism, furthering the field’s advancement^25^. Quantifiable performance increases of 1.91% in mIoU and 1.19% in Frequency Weighted Intersection over Union (FWIoU) were achieved by this novel technique, which focused on improving model attention on important crop and weed categories. The model’s improved performance in challenging field settings shows how useful it is in actual agricultural scenarios in which environmental influences can have a big influence on identification accuracy.

### Integration of knowledge distillation and real-time optimization

Researchers are investigating knowledge distillation approaches in conjunction with conventional image processing methods as a result of the desire for real-time agricultural monitoring. One important research, which focused on maize segmentation applications, combined the DeepLabV3 + architecture with traditional image processing methods and knowledge distillation frameworks^26^. The crucial problem of preserving high accuracy while attaining real-time speed and cutting computational overhead through model size optimization was effectively handled by this hybrid technique.

### Analysis of segmentation architectures in specific crop environments

Research on paddy field applications has shed important light on how well various segmentation frameworks perform in comparison. Extensive comparison analyses shown that in rice field environments, Pyramid Scene Parsing Network (PSPNet) continuously outperformed both UNet and SegNet designs, attaining mIoU values in the 70%–80% range^14^. The significance of choosing suitable designs depending on particular crop traits and field circumstances is shown by this performance difference. The development of UNet architectures has produced complex variations with cutting-edge training techniques. With mIoU values as high as 92.34%, a UNet++ implementation with deep supervision techniques demonstrated exceptional performance in sugar beet field applications^31^. This architecture showed the benefits of architectural improvements and sophisticated training approaches in agricultural segmentation tasks by considerably outperforming baseline UNet++ implementations as well as classic UNet models.

In agricultural applications, the combination of object detection and semantic segmentation approaches has demonstrated encouraging outcomes. A rich Sugarbeets dataset with 1,300 images was used to train a hybrid model that combined semantic segmentation with YOLO (You Only Look Once) object recognition^32^. The potential advantages of merging complementing computer vision techniques for improved agricultural monitoring were demonstrated by this integrated system, which achieved 93% accuracy.

Agricultural imaging frequently takes place in difficult settings, especially when drone-based systems are being used, which can cause motion blur. Researchers created the customized architecture DeBlurWeedSeg to deal with motion-blurred drone footage after realizing this constraint^33^. A major practical issue in aerial agricultural monitoring systems was resolved by this creative method, which much outperformed traditional segmentation algorithms without deblurring capabilities.

Semantic segmentation performs well for weed-crop identification across different crops; mIoU values of up to 92% and F1-scores of over 90% have been observed. Custom designs such as DeBlurWeedSeg, ERFNet with dual-loss, UNet++ with deep supervision, and U-Net based on Inception-ResNetV2 improve accuracy and robustness while addressing real-world issues. Lightweight models and knowledge distillation enable real-time performance, while hybrid approaches improve practical utility. The quality and diversity of datasets are crucial for better learning and broader generalizability. The development of automated, accurate, and effective crop monitoring systems has advanced significantly with these developments in semantic segmentation for agricultural applications. Through more precise and timely weed-crop distinction capabilities, the technology’s ongoing development promises to improve crop production projections, maximize resource management, and advance sustainable agricultural methods.

Despite the outstanding F1-scores and mIoU metrics that contemporary deep learning models, such U-Net++, DeepLabV3+, and PSPNet, show across a variety of crops and environments, the majority of methods have some significant drawbacks. Without explicit edge modeling, it is still difficult to distinguish between weeds and crops. Additionally, most research rely on single-field or crop-specific data, making it uncommon to examine generality across datasets or geographic regions. Also neglected are post-processing methods that may greatly improve fine-grained segmentation, such as CRF. By combining an edge detection module, boundary-aware loss to sharpen object edges, using CRF-based post-refinement to lower segmentation noise, and verifying generalization ability across datasets, our suggested PSPEdgeWeedNet directly tackles these issues. Combined, these enhancements address the significant flaws in earlier research and help create more accurate semantic segmentation systems for practical agricultural monitoring.

### Multispectral and NIR-based approaches in agricultural segmentation

Multispectral and near-infrared (NIR) data have been used more and more in precision agriculture in recent years to increase the accuracy of crop and weed segmentation. The pioneering public dataset RafanoSet^34^ includes 85 multispectral images of fields of Triticum aestivum that include Raphanus raphanistrum. The images are annotated at the pixel level to locate weeds. The images, which were taken in 17 different scenarios and contain spectral bands including Blue, Green, Red, NIR, and RedEdge, provide a wealth of information for segmentation model training and evaluation. An improved SegFormer architecture was presented in a related effort to use UAV-based images to extract spatial patterns in agricultural landscapes^35^. With 98.42% pixel accuracy and 96.91% IoU, the model’s integration of Efficient Channel Attention, BiFPN layers, and MLP-based modules enhances feature extraction and yields noteworthy results, which are especially advantageous for small-scale farms and field borders. Furthermore, as an alternative to conventional data augmentation in crop-weed segmentation tasks, a conditional GAN (cGAN) was used in another work to create realistic multispectral images, encompassing RGB and NIR channels^36^. In addition to improving visual realism, using synthetic data increased semantic segmentation network’s accuracy. Additionally, hyperspectral imaging has demonstrated potential; in one research, five weed species and soybean plants were classified using a robotic hyperspectral scanning platform in a greenhouse setting^37^. The study highlighted unique chemical fingerprints among species and obtained good classification accuracy by using 983 hyperspectral cubes and Savitzky–Golay derivative preprocessing. Additionally, early identification of the parasitic weed known as sunflower broomrape was accomplished with hyperspectral imaging, with classification accuracies of 76% and 89% at 31 and 38 days after planting, respectively^38^. The study illustrated the potential of hyperspectral methods for prompt, site-specific weed treatment by examining certain leaf segments. When taken as a whole, these studies highlight how multispectral and hyperspectral imaging are increasingly helping to advance precision farming by improving plant discriminating and detecting weeds early.

## Materials and methods

### Dataset

The peanut (groundnut) dataset employed in this study was sourced from a publicly available GitHub repository^39^ and is specifically designed to support research in crop-weed segmentation. It comprises 400 high-resolution RGB images, each with a resolution of 720 × 960 pixels, captured from real-world peanut fields located near Da Nang, Vietnam. These images provide a rich and diverse visual representation of peanut crops alongside several common weed species, most notably goose grass and nut grass.

The dataset is annotated with two semantic classes: crop (representing peanut plants) and weed (aggregating all types of invasive species). The annotation process was meticulously performed under the consultation of domain experts in agriculture, ensuring high-quality ground-truth labels. On average, each image required around 20 minutes to annotate due to the intricate and detailed nature of the scenes.

**Fig. 2:**
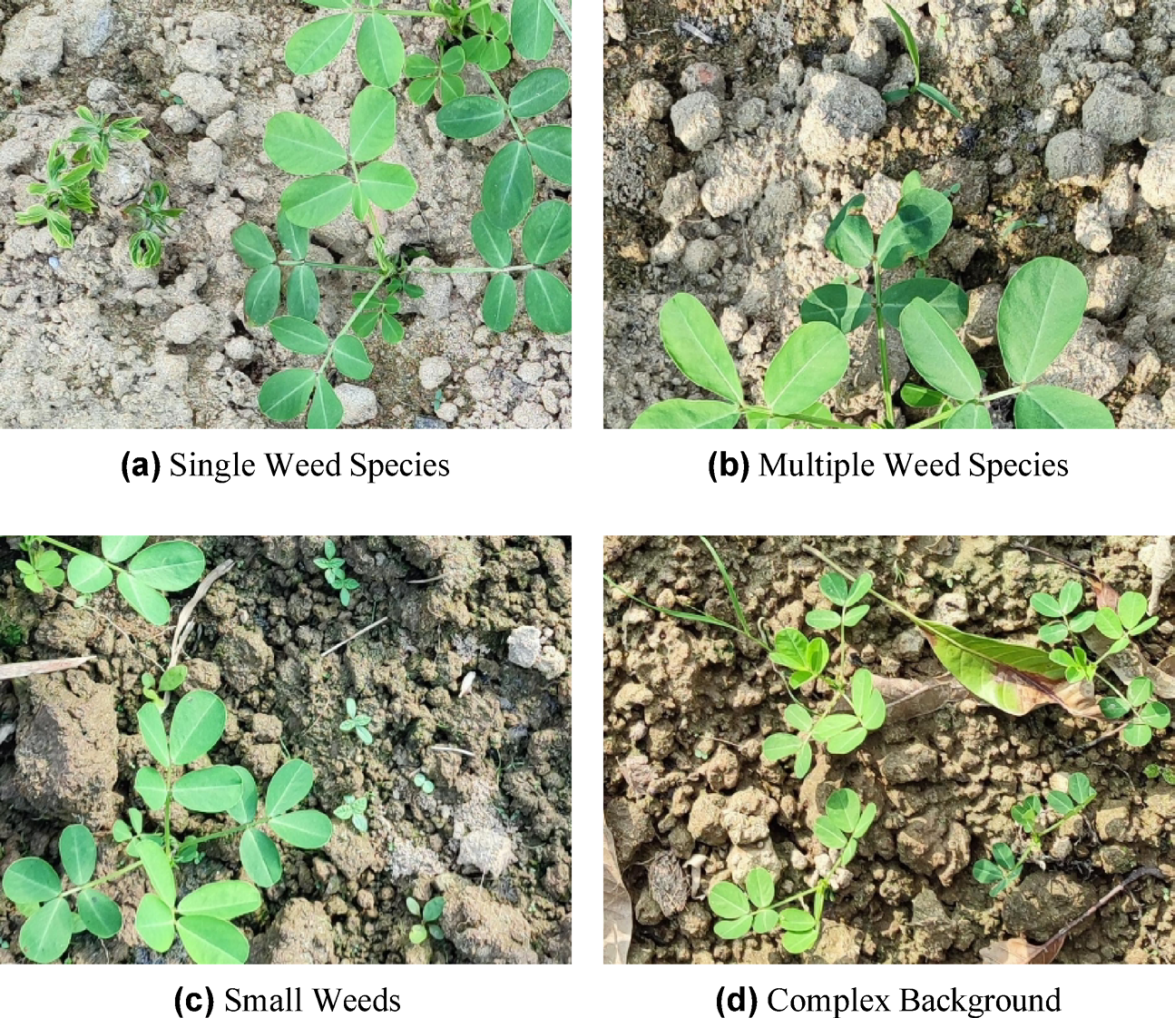
A peanut example dataset is used to illustrate the dataset’s diversity and recognition challenges. The images depict a range of field scenarios, including single, tiny, and mixed weed species, as well as crops and weeds with intricate backgrounds. These illustrate the challenges of weed detection technologies by showcasing the seemingly complicated and identical backgrounds of numerous weed types^39^.

Figure 2 illustrates the peanut dataset showcasing diverse and complex weed scenarios, emphasizing the challenges in accurate weed detection.

**Fig. 3:**
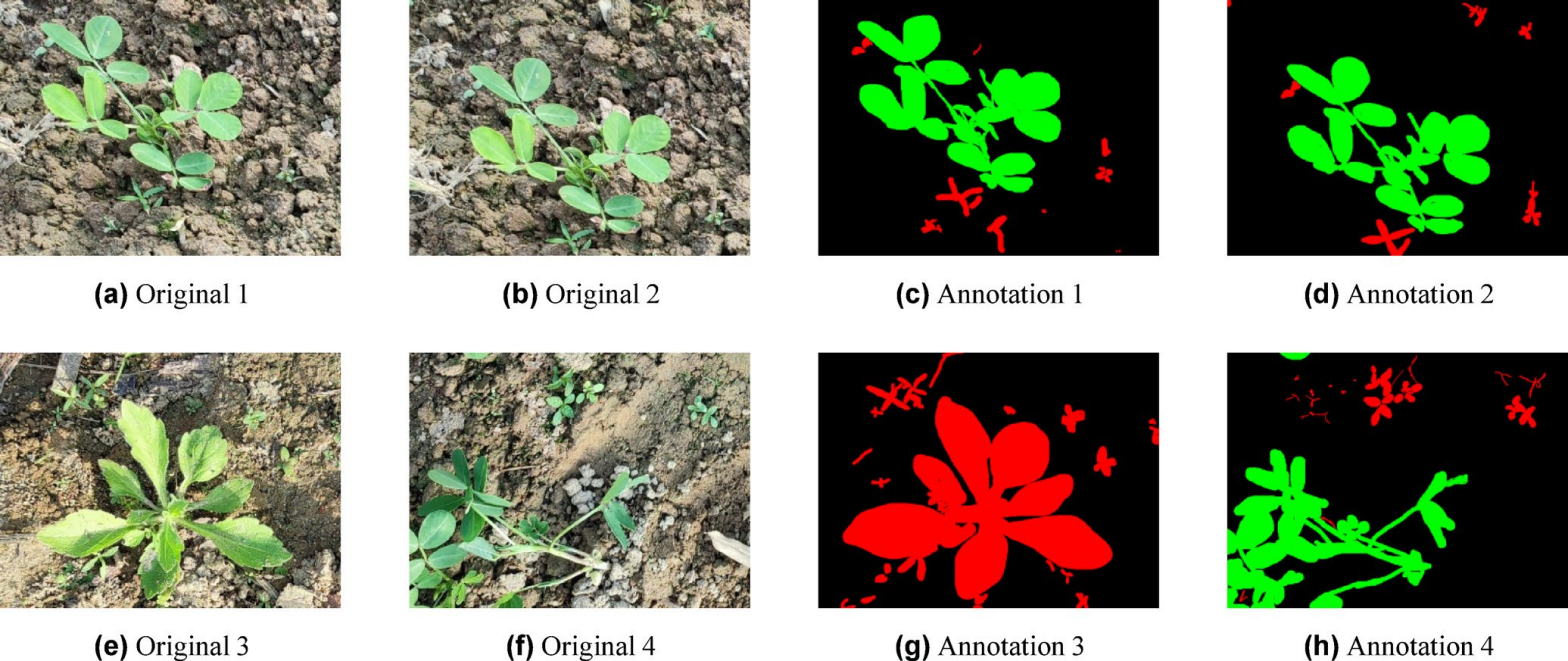
Representative samples from the dataset: (left) original RGB image and (right) corresponding annotated image, where green denotes crop regions and red indicates weed areas^39^.

This dataset presents several complex segmentation scenarios that are representative of actual field conditions. These include the occurrence of small-scale weed patches, inter-plant occlusions, and large differences in plant size, all of which greatly complicate accurate segmentation. Specifically, the visual resemblance and dense spatial distribution of weeds and crops, as well as background clutter, necessitate models that can capture contextual cues and fine-grained limits. The peanut dataset represents a rigorous standard for assessing the efficacy and resilience of contemporary Deep Learning-based semantic segmentation models in precision agriculture because of these features. Sample original and annotated images from the dataset are shown in Figs. 3.

### Experimental setup

The experiments in this study were conducted on a computational platform equipped with an NVIDIA A100 GPU. The experimental environment utilized the PyTorch framework, running on a Microsoft Windows 11 system with 8 GB of RAM. The platform supported CUDA 11.8, enabling efficient GPU acceleration for Deep Learning tasks. All experiments were implemented using Python 3.10.12.

### Training parameters

To enhance model performance, the training parameters were systematically fine-tuned through multiple experimental iterations. A resolution of 600 × 600 pixels was selected for the input images, as it provides an optimal balance between computational efficiency and the retention of key image features. While smaller image sizes could result in the loss of crucial information, larger sizes would unnecessarily increase the computational load without offering significant performance benefits. The number of epochs was set to 100 to ensure sufficient learning while avoiding overfitting.

The model’s performance was optimized using an Adam optimizer with a learning rate of 1e-4 and a batch size of 16. Using a pretrained ResNet101 backbone, the model architecture used a custom PSPNet with edge detection included. Dense CRF was used as a post-processing step to further improve the visualization of segmentation findings. By successfully resolving minor errors and enhancing boundary accuracy, this method, when used for visualization purposes after inference, provides cleaner segmentation boundaries.

Effective feature extraction is made possible by using a pretrained Convolutional Neural Network (CNN) as the backbone. This promotes quicker convergence and improved performance with less data. To balance the pace of convergence and minimize overfitting, the learning rate was carefully tuned during training. The dataset was randomly split into 80% for training, 10% for validation, and 10% for testing. Although the split was random and not stratified, class-weighted loss functions were used to address class imbalance across the three semantic classes—Crop, Weed, and Background. Specifically, class weights of 2 for Crop, 3 for Weed, and 1 for Background were applied to ensure that minority classes (Crop and Weed) had a greater influence during model optimization. Since the usage of augmentation techniques such random rotations, color jitter, and horizontal and vertical flips resulted in lower metric values, including decreased accuracy, the model trained without data augmentation was taken into consideration. Furthermore, in contrast, the visuals of predictions were substantially worse with data augmentation. This might be explained by the model’s inability to generalize well after being trained on sparse data with augmentation, which resulted to noisy predictions and imprecise limits. The model’s ability to concentrate on the dataset’s essential characteristics without augmentation resulted in more consistent and precise performance. A boundary-aware loss was used to improve edge detection performance, and the Cross-Entropy Loss function was used for the segmentation task, with weighted class contributions to rectify the class imbalance.

The model contains a total of 48,271,428 trainable parameters, which are learned during the training process. This large number of parameters indicates an architecture capable of capturing intricate patterns in the data, allowing the model to potentially learn highly detailed features.

### Evaluation metrics

This research uses important evaluation metrics, including as training and validation accuracy, precision, recall, F1 score, and Intersection over Union (IoU), to thoroughly assess the model’s effectiveness and performance in semantic segmentation tasks.

- Accuracy: Accuracy measures the number of pixels correctly classified as weeds or crops.
- Precision: Precision quantifies the number of true positive cases that a model predicts, or the accuracy of the model’s positive predictions.
- Recall: The frequency with which a model accurately detects positive examples (true positives) out of all the actual positive samples in the dataset is known as recall.
- F1 score: It is the harmonic mean of precision and recall, giving a fair assessment of a model’s precision (the capacity to detect positive occurrences) and recall (the ability to minimize false positives).
- IoU: A metric used to evaluate the accuracy of object detection models and segmentation models.
- Confusion Matrix: In semantic segmentation, a confusion matrix is used to evaluate how well a model classifies each pixel into the correct class.
- Visualization: Visualizing segmentation results alongside the ground truth helps understand where the model makes errors, particularly in boundary definitions and tiny object detection.

## Proposed model—PSPEdgeWeedNet

**Fig. 4:**
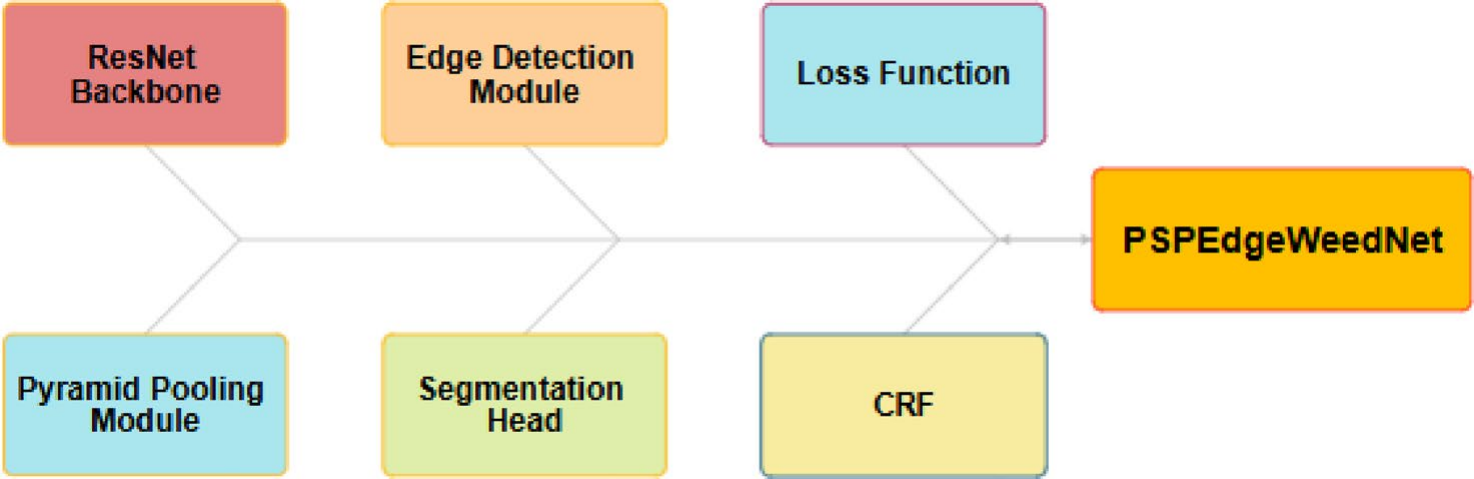
Basic blocks used in proposed work.

Figure 4 depicts basic blocks used in the proposed work.

The proposed model PSPEdgeWeedNet is a modified version of PSPNet (Pyramid Scene Parsing Network), specifically tailored for semantic segmentation tasks in agriculture, such as accurately distinguishing between crops and weeds. It utilizes ResNet101 as the backbone, a deep convolutional neural network pretrained on ImageNet, which effectively extracts rich feature representations while preserving spatial details essential for segmentation. To enhance contextual understanding, a pyramid pooling module (PSP block) is integrated, enabling the model to capture information at multiple scales. This is particularly valuable for agricultural images, where objects may appear in varied sizes and complex backgrounds are common. An additional edge detection module is incorporated into the architecture to improve the delineation of object boundaries.

This component learns edge features from the same feature maps used for segmentation and generates an edge map to guide the model in identifying class borders more precisely. To reinforce this behavior during training, a custom Boundary Loss is introduced. It applies a Sobel operator to both the predicted segmentation output and the ground truth mask to extract edge information and then computes the mean squared error between them. In conjunction with the conventional CrossEntropyLoss, this boundary-aware loss encourages more precise boundary predictions and lessens class overlap. To improve the visual depiction of the segmentation precision, a Conditional Random Field (CRF) is also used as a post-processing step during the visualization phase to maintain fine details and sharpen boundaries.

In summary, a dual-loss approach, edge detection for boundary refinement, and pyramid pooling for global context work together to produce more precise and thorough segmentation. This is particularly useful in agricultural settings where accurate and contextually aware segmentation is necessary due to the minor variations between weeds and crops.

**Fig. 5:**
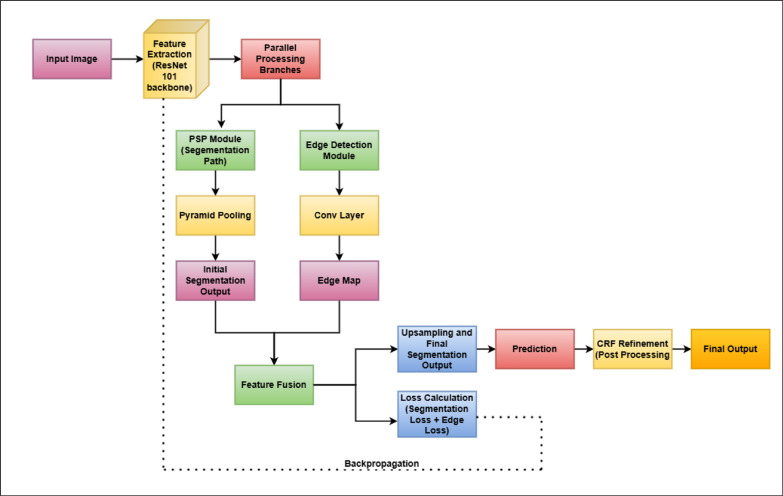
Proposed PSPEdgeWeedNet block diagram.

This approach ensures that both segmentation accuracy and edge refinement are optimized. Figure 5 illustrates the block diagram of the proposed PSPEdgeWeedNet model.

### Edge detection module and boundary aware loss

By including an edge detection module, the proposed PSPEdgeWeedNet enhances localization in high-resolution agricultural images by learning semantic areas such as crop, weed, and background. The module adds an edge detection head for the convolutional layer and pulls feature maps from the ResNet101 backbone. This head highlights changes between semantic classes by learning a 1-channel edge probability map. Edge prediction is guided by using a unique boundary-aware loss based on Sobel edge detection.

To guarantee distinct, precise object borders and oversee edge prediction, the Boundary-aware Loss technique is employed. By extracting gradient magnitude from both ground truth and anticipated segmentation maps using Sobel filters and minimizing the difference using Mean Squared Error, it ensures clear and well-aligned borders in challenging agricultural images.

Boundary-aware loss implementation works by combining traditional semantic segmentation loss with a specialized boundary detection loss to enhance segmentation accuracy at class boundaries. It extracts edge information from predicted segmentation maps and ground truth labels using Sobel edge detection filters. The Sobel operator applies two 3 × 3 convolution kernels to compute gradient magnitudes, highlighting boundary regions. The edge strength is calculated as the square root of the sum of squared gradients in both directions, creating edge maps emphasizing transitions between different classes like crops, weeds, and background. The boundary loss is computed as the Mean Squared Error (MSE) between the predicted edge map and the ground truth edge map, measuring the alignment of the model’s predicted boundaries with the actual class boundaries in the labeled data. This loss function optimizes for correct pixel-wise classification and accurate boundary delineation. By penalizing boundary misalignment, the model learns to pay special attention to boundary regions where segmentation errors are most visually apparent and functionally important, particularly in crop/weed segmentation tasks. This dual-objective approach helps the model achieve better overall segmentation performance by ensuring that class transitions are sharp and well-defined, rather than just focusing on broad regional classification accuracy.

#### Boundary-aware loss function

To enhance the precision of object boundaries in semantic segmentation, the authors introduce a *boundary-aware loss* based on Sobel edge detection. This loss penalizes differences between the predicted and ground truth edge maps.

*Predicted Segmentation* Let the network output be a raw score tensor:

1 $$P(x) \in {\text{R}}^{H \times W \times C}$$

where *H*, *W* , and *C* denote the height, width, and number of classes, respectively.

The predicted class label for each pixel is obtained via:

2 $$\hat{Y}(x) = \mathop {{\text{argmax Softmax}}(P(x)) }\limits_{c}$$

where $\hat{Y}(x) \in \{ 0,{1},...,C - {1}\}^{{^{H \times W} }}$ is the class prediction map.

*Ground Truth Segmentation* Let the ground truth label map be:

3 $$Y(x) \in \{ 0,{1},...,C - {1}\}^{{^{H \times W} }}$$

*Sobel Edge Detection* To extract edge information, we apply Sobel operators in both horizontal (*S*_*x*_) and vertical (*S*_*y*_) directions:

4 $$S_{x} = \begin{array}{*{20}c} { - {1}} & 0 & {1} \\ { - {2}} & 0 & {2} \\ { - {1}} & 0 & 1 \\ \end{array} \;S_{y} = \begin{array}{*{20}c} { - 1} & { - 2} & { - 1} \\ 0 & 0 & 0 \\ 1 & 2 & 1 \\ \end{array}$$

The edge maps of the predicted and ground truth masks are calculated as:

5 $$E_{{\hat{Y}}} = \sqrt {(S_{x} * \hat{Y})^{{2}} + (S_{y} * \hat{Y})^{{2}} }$$

6 $$E_{Y} = \sqrt {(S_{x} * Y)^{{2}} + (S_{y} * Y)^{{2}} }$$

where ∗ denotes 2D convolution, and $E \hat{Y},E_{Y} \in {\text{R}}^{{^{H \times W} }}$ represent the edge magnitude maps of the prediction and ground truth*.*

*Boundary-aware Loss* The loss is defined as the mean squared error between the predicted and ground truth edge maps:

7 $$L_\textrm{boundary} =\frac{1}{H \times W} \sum\limits_{i = 1}^{H} \sum\limits_{j = 1}^{W} {(E \hat{Y}(i,j) - E_{Y} (i,j))^2} $$

This encourages the predicted segmentation boundaries to align closely with those in the ground truth.

**Fig. 6:**
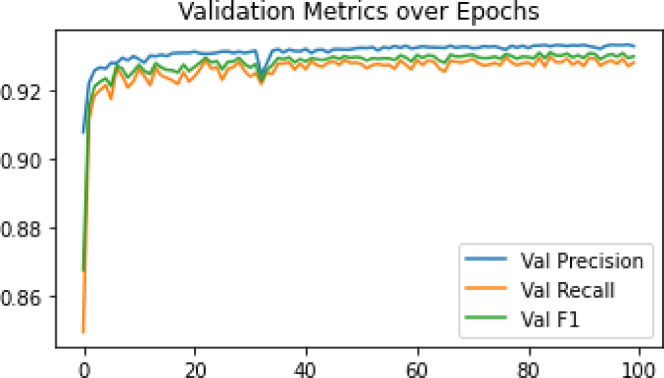
Validation metrics over epochs in PSPEdgeWeedNet.

*Total Loss* The boundary-aware loss is used in conjunction with the standard cross-entropy loss:

8 $$L_{{{\text{total}}}} = L{_\text{CE}} + \lambda \cdot L_{{{\text{boundary}}}}$$

where *L*_CE_ is the pixel-wise cross-entropy loss, and *λ* is a weighting factor controlling the influence of boundary supervision. Based on the performance metrics obtained after training the models and evaluating them on the validation dataset, it was observed that PSPNet with class weights set to crop = 2, weed = 3, and background = 1 (c = 2, w = 3, b = 1) achieved superior results compared to other class weighting strategies. Furthermore, integrating a boundary-aware loss function into PSPNet led to noticeable improvements in segmentation accuracy, precision, recall, and F1-score when compared to the baselinePSPNet model. Building on these findings, a novel model, PSPEdgeWeedNet, was proposed. This model retained the optimal class weights (2, 3, 1) and incorporated both the boundary-aware loss and an additional edge detection module to explicitly refine object boundaries. The inclusion of the edge detection branch further enhanced the segmentation performance across all evaluation metrics, including per-class Intersection over Union (IoU), overall precision, recall, and F1-score as shown in Fig. 6. The trend of validation metrics across epochs indicates a consistent improvement in precision, recall, and F1 scores throughout the training process. However, a slight dip in all three metrics is noticeable around the 30th to 35th epochs, which may be attributed to temporary model instability or overfitting fluctuations. Following this phase, the metrics recover and continue to improve, ultimately reaching approximately 93%, indicating strong generalization performance in later epochs.

**Fig. 7:**
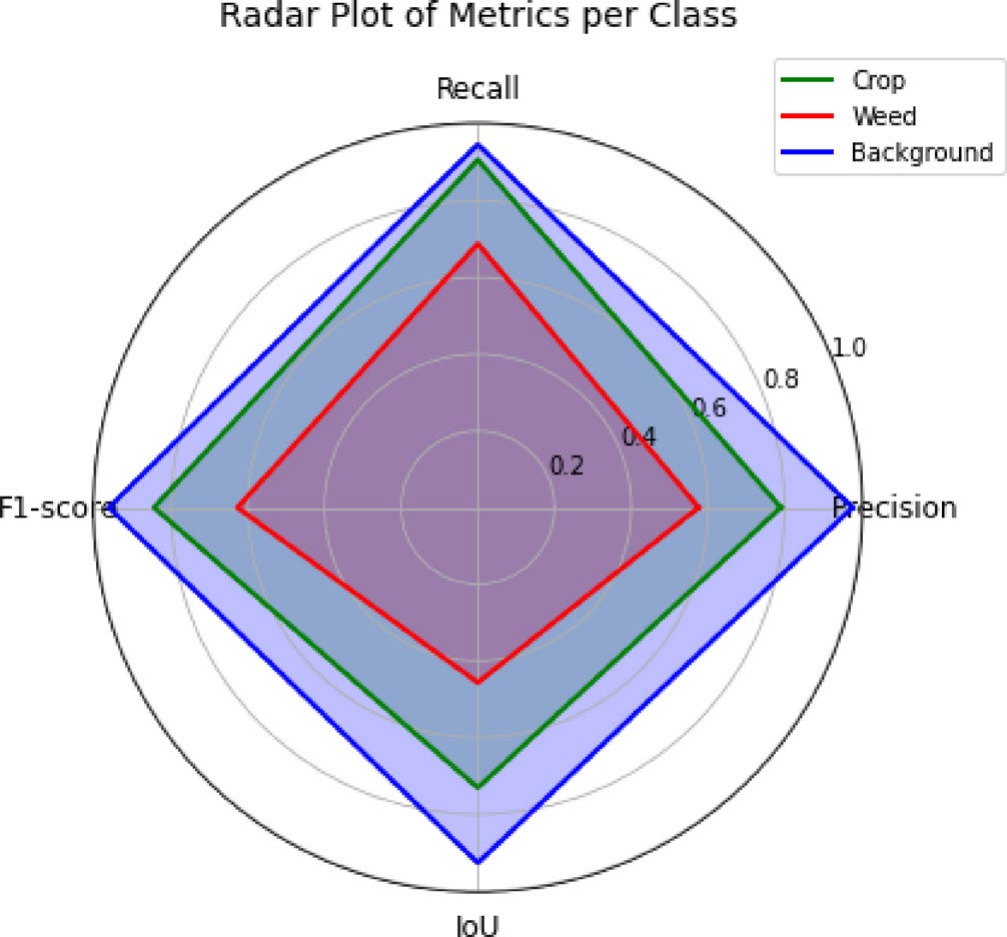
Radar chart using the PSPEdgeWeedNet model that shows the IoU, F1 score, recall, and precision for each class.

Figure. 7 illustrates the radar chart comparing precision, recall, F1 score, and IoU across each class in the proposed model. The background class consistently achieves the highest scores across all metrics, followed by the crop class. The weed class ranks lowest; however, it still attains approximately 60% in both precision and F1 scores, and nearly 70% in recall, indicating reasonable detection performance.

Conditional Random Fields (CRF) were applied as a post-processing refinement technique during the visualization phase. CRF significantly enhanced the delineation of crop and weed boundaries and reduced inter-class misclassifications, especially in overlapping regions. Although the model occasionally missed very small or sparse weed instances, the overall boundary precision in visual outputs was markedly improved, demonstrating the effectiveness of the integrated architecture and post-processing strategy.

**Fig. 8:**
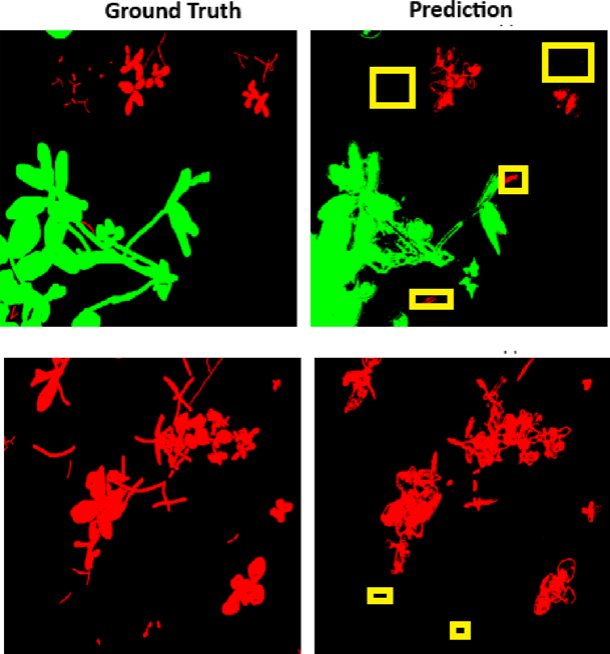
Visualization of ground truth and prediction where the proposed model PSPEdgeWeedNet failed. Green color represents crop, red color for weed, and black color for the background. Misclassifications and missed detections are highlighted using yellow boxes.

The Fig. 8 shows the visualization of ground truth and prediction where the proposed model failed to detect the smaller instances of weed. It shows that despite its excellent performance, the proposed PSPEdgeWeedNet model had trouble identifying microscopic or tiny weed instances, especially in situations where the weeds were partially obscured or sparsely scattered. In the segmentation result, these smaller weeds were either completely ignored or incorrectly categorized as background. Reduced saliency of fine-scale information in deeper network layers and possible loss of spatial resolution during the encoding process are the causes of this problem. Deep segmentation networks frequently face the difficulty of identifying tiny objects, particularly when the item size is smaller than the network’s receptive field. The fine-grained patterns linked to smaller weeds might not have been adequately captured by the model’s edge refinement and pyramid pooling techniques.

**Fig. 9:**
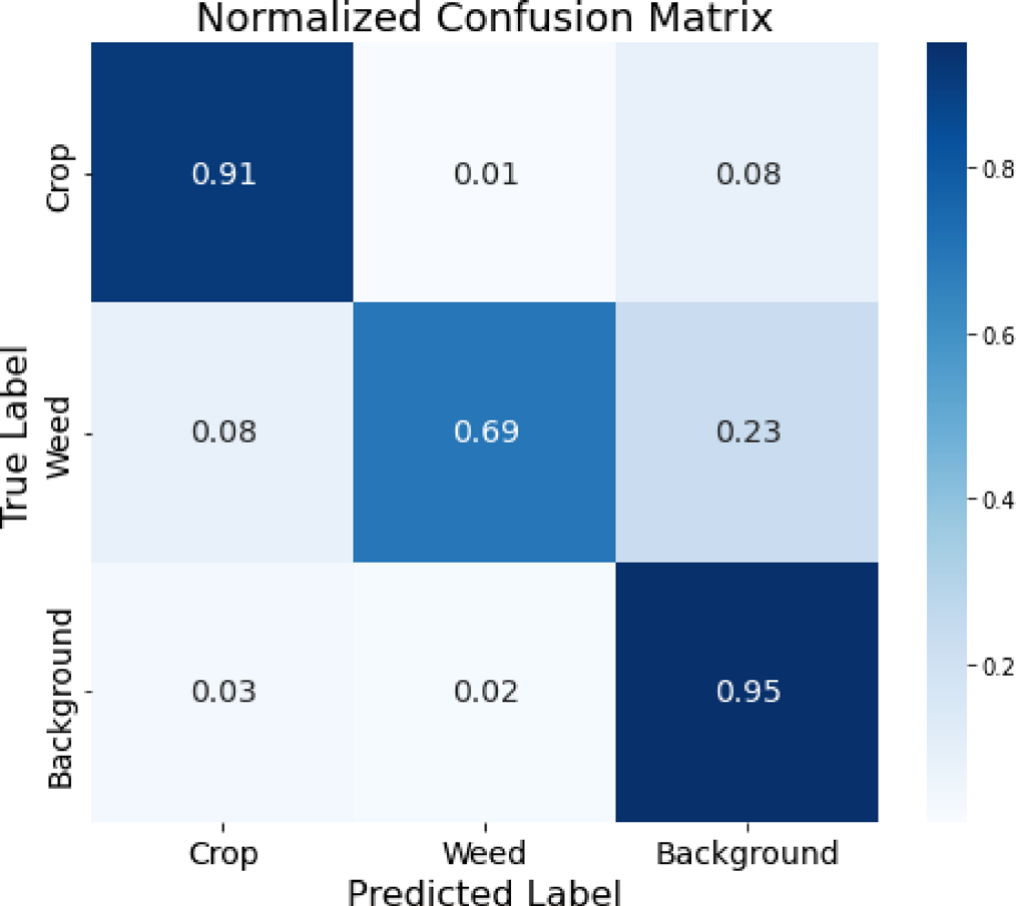
Confusion matrix of proposed model PSPEdgeWeedNet.

The model’s ability to differentiate between the three classes, crop, weed, and background, is demonstrated by the normalized confusion matrix as shown in Fig. 9. With a high true positive rate of 91%, the model has a remarkable capacity to recognize crop pixels, whereas just 1% are misclassified as weed and 8% are misclassified as background. The visual elements of weed and the other two classifications overlap, as seen by the fact that weed pixels are properly categorized 69% of the time, although there is a noticeable misunderstanding with background (23%), and 8% are misclassified as crop. With a 95% classification accuracy, the background class shows the least amount of misunderstanding with crop (3%) and weed (2%). Table 2 presents a comparative analysis of PSPNet, PSPEdgeWeedNet without CRF, and PSPEdgeWeedNet with CRF. The results indicate that the integration of CRF in the proposed PSPEdgeWeedNet significantly enhances boundary delineation for both weeds and crops, and effectively preserves leaf structures. Moreover, the model demonstrates zero misclassification, thereby contributing to an overall improvement in segmentation quality.

**Table 2:** Comparison of PSPNet and PSPEdgeWeedNet variants.

Aspect	PSPNet	PSPEdgeWeedNet without CRF	PSPEdgeWeedNet with CRF
Boundary definition	Poor; boundaries between crops and weeds are blurred	Improved boundary sharpness compared to PSPNet	Well-defined and continuous boundaries
Weed leaf structure preservation	Inadequate; leaf structures are not clearly captured	Partial preservation; leaf edges are still not sharply structured	Good preservation; leaf morphology is maintained
Misclassification	Present; crops misclassified as weeds	Minimal; misclassifications largely eliminated	No significant misclassification observed
Edge sharpness	Low; edges appear soft and diffused	Moderate; improved but not optimal	High; edges are sharp and detailed
Tiny weed detection	Missed frequently	Slight improvement, but still limited	Some very small weeds missed, but overall better retention
Overall segmentation quality	Suboptimal	Moderate; better than baseline	Significantly improved

### Limitations and generalization capability

**Fig. 10:**
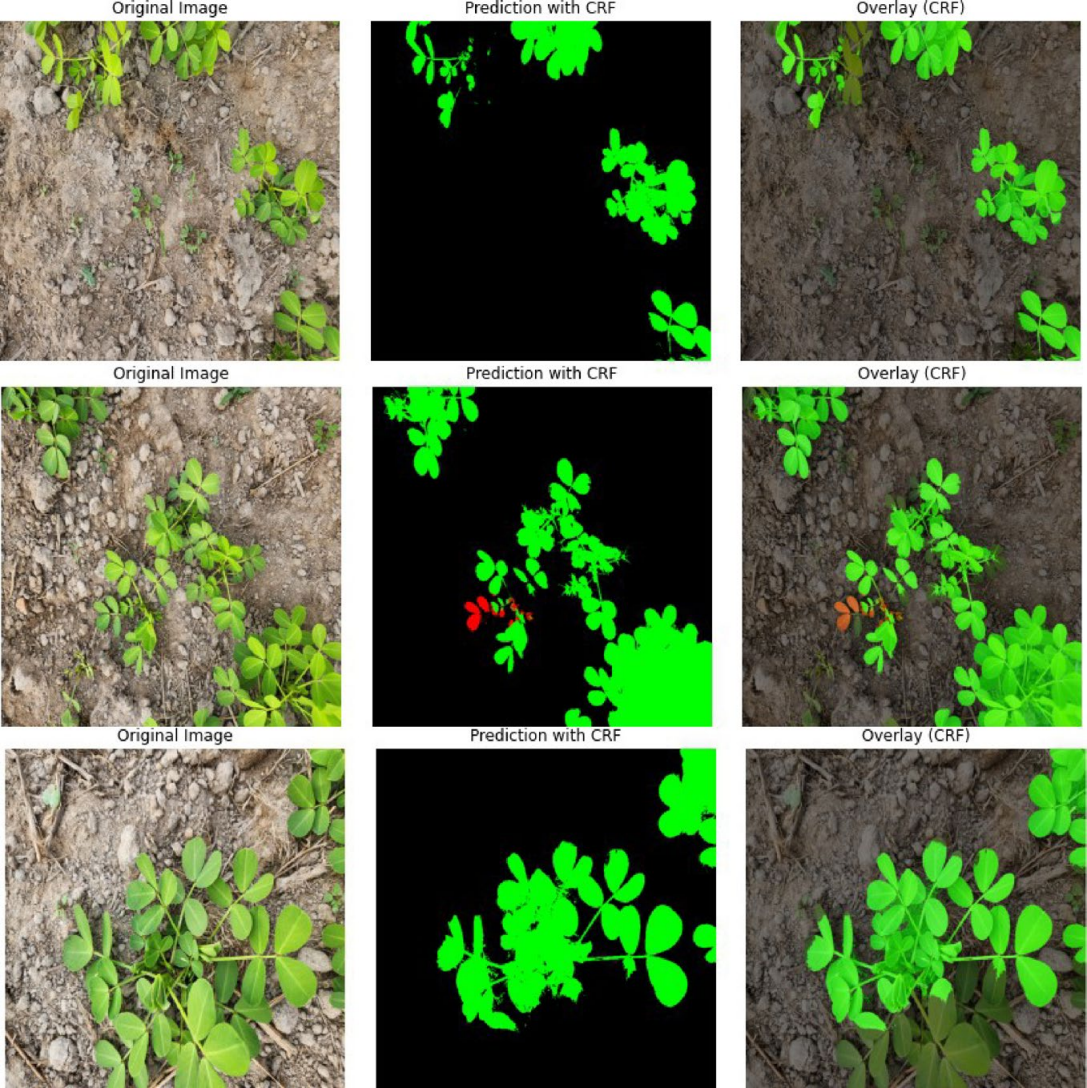
Visualization of original image, prediction, and overall of PSPEdgeweedNet on a new dataset collected from the Udupi region of Karnataka, India.

One of the key limitations of this study lies in the relatively small size and geographic scope of the training dataset, which comprises only 400 images of peanut crops collected from a single region. Such a dataset may not fully capture the variability in field conditions, weed species, or lighting scenarios encountered in other agricultural environments. To evaluate the model’s generalizability, we tested the trained network on an independent groundnut dataset collected from the Udupi region of Karnataka. This dataset contains a diverse range of weed species, including different types of broadleaved weeds and grasses, and was captured under varying lighting conditions. Without any additional retraining or fine-tuning, the model was able to correctly identify groundnut crops and detect certain broadleaved weeds and grasses in several samples, demonstrating a level of robustness and transferability. However, the model struggled to recognize other types of broadleaved weeds, indicating limitations in its ability to generalize to all unseen weed variants. The Udupi dataset was utilized solely for visualization purposes, showcasing side-by-side comparisons of the original image, predicted segmentation map, and overlay to highlight both accurate detections and notable failure cases. Figure 10 illustrates the visualization of the original image, the model’s prediction, and the overlay between them. The figure demonstrates that the model successfully identifies nearly all peanut crops, even without being retrained on this new dataset from a different region. However, it struggles to detect weeds, as the weed types in this area were not included in the training data, making them unfamiliar to the model. Despite this, the model still performs well in accurately recognizing the crops.

## Comparison with other semantic segmentation models

The performance comparison across various DL models, as shown in Table 3 for semantic segmentation, reveals that the proposed PSPEdgeWeedNet model significantly outperforms all other architectures. It achieves the highest scores across all evaluation metrics, with a precision of 0.9399, a recall of 0.9335, F1-score of 0.9358, an IoU of 0.8797, and a competitive accuracy of 0.9335. This indicates that the proposed model not only detects relevant classes more accurately but also handles boundary and overlap regions more effectively. Among the baseline models, DeepLabv3 shows strong overall performance (F1-score of 0.8329 and IoU of 0.69), while SegNet and UNet also perform reasonably well. Swin-UNet, though achieving the highest accuracy (0.9511), falls behind in F1-score and IoU, suggesting that it might be biased toward the dominant class. Accuracy alone can be deceptive in semantic segmentation tasks such as crop-weed identification because of class imbalance, where background pixels frequently take up the majority of the image. Measures such as Intersection over Union (IoU), precision, recall, and F1 score provide more accurate and insightful evaluations. By reducing false positives, precision assesses how accurate positive predictions are, whereas recall concentrates on the model’s capacity to catch all pertinent cases. The F1 Score balances precision and recall, providing a single measure of accuracy that accounts for both types of errors. IoU quantifies the spatial overlap between predicted and actual segmentation, making it valuable for assessing object boundaries and region-based accuracy. Together, these metrics provide a comprehensive evaluation framework that better captures the effectiveness and robustness of segmentation models in detecting and delineating agriculturally significant features. By balancing precision and recall, the F1 Score offers a single accuracy metric that takes into consideration both kinds of errors. IoU is useful for evaluating object boundaries and region-based accuracy since it measures the spatial overlap between expected and actual segmentation. When combined, these metrics offer a thorough assessment methodology that more accurately depicts the efficiency and resilience of segmentation models in identifying and defining traits of agricultural importance. So it can be concluded that the proposed PSPEdgeWeedNet model outperforms all the baseline semantic segmentation models evaluated in this study. Although PSPEdgeWeedNet exhibits a slightly lower inference speed (86 FPS) compared to other models like UNet (166 FPS) and SegNet (145 FPS), it outperforms them in all other key performance metrics such as accuracy, mIoU, F1-score, and boundary delineation. This makes the proposed model a more suitable choice for applications where segmentation quality is prioritized over raw inference speed.

**Table 3:** Performance comparison of different models for semantic segmentation (Significant values are highlighted in bold).

Model	Precision	Recall	F1 Score	IoU	Accuracy	Inference Speed
UNet	0.7942	0.8053	0.8177	0.6887	0.9327	166 FPS
SegNet	0.8269	0.7720	0.7866	0.6802	0.9345	145 FPS
PSPNet	0.6631	0.7257	0.6756	0.5553	0.8777	95 FPS
DeepLabv3	0.8162	0.8537	0.8329	0.6900	0.9481	89 FPS
Swin-UNet	0.7829	0.8040	0.7925	0.6827	**0.9511**	98.92 FPS
Lightweight ViT-based Model	0.6029	0.5823	0.5848	0.4708	0.8428	150 FPS
Proposed PSPEdgeWeedNet	**0.9399**	**0.9335**	**0.9358**	**0.8797**	0.9335	86 FPS

**Fig. 11:**
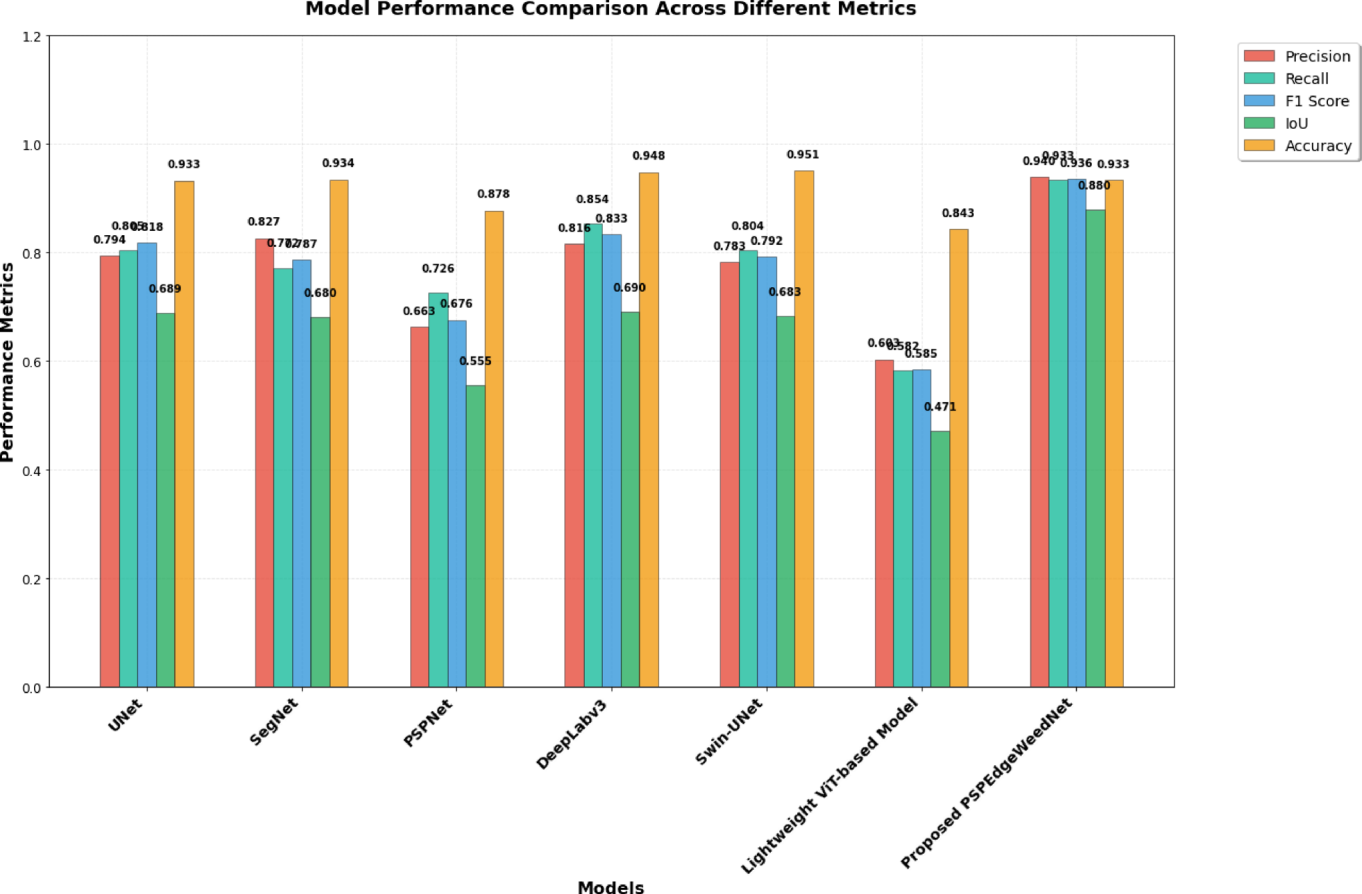
Performance comparison of semantic segmentation models.

Figure. 11 presents a bar plot comparing the performance of various semantic segmentation models across multiple evaluation metrics, including precision, recall, F1 score, IoU, and accuracy.

**Fig. 12:**
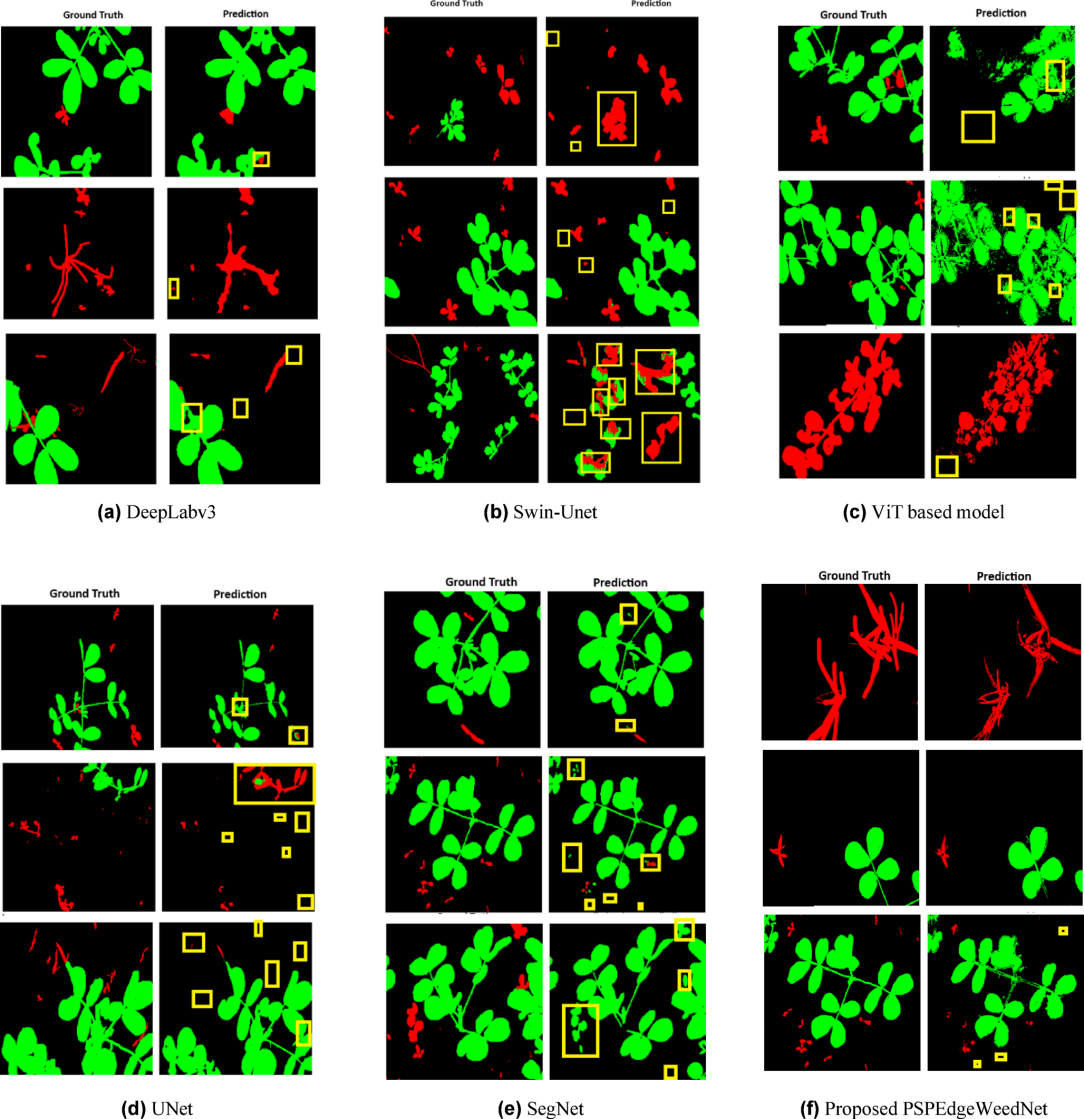
Visualization of ground truth and corresponding predictions (CRF refined) generated by different semantic segmentation models. Green color represents crop, red color for weed, and black color for the background. Misclassifications and missed detections are highlighted using yellow bounding boxes.

Visualization of ground truth and corresponding predictions generated by various semantic segmentation models, post- processed with Conditional Random Fields (CRF) for boundary refinement, are shown in Fig. 12. Each row displays side-by-side comparisons of the input image, ground truth annotation, and CRF-refined predicted mask. Yellow bounding boxes are overlaid to highlight regions of misclassification (e.g., crop predicted as weed or background) and missed detections (e.g., weeds not detected). These visualizations demonstrate the capability of each model to localize and classify crop, weed, and background regions accurately, as well as the effectiveness of CRF refinement in enhancing boundary precision and reducing spatial ambiguity.

The comparison of UNet, PSPNet, SegNet, DeepLabV3, Swin-Unet, a lightweight transformer model based on ViT, and PSPEdgeWeedNet is presented in Table 4, which concludes that PSPEdgeWeedNet provides the optimum performance, accuracy, and efficiency balance, especially for tasks requiring boundary localization and fine-grained segmentation.

**Table 4:** Comparison of segmentation models.

Model	Pyramid pooling	Edge detection focus	Boundary precision	Computational efficiency	Key strengths
UNet	✗	✗	Moderate	High	Strong performance in small datasets
SegNet	✗	✗	Moderate	Moderate	Memory-efficient decoding
PSPNet	✓	✗	Moderate to High	Moderate	Multiscale context understanding
DeepLabv3	✓	✗	Moderate to High	Moderate to Low	Captures large context without resolution loss
Swin-Unet	✓	✗	High	High	Good for dense prediction tasks
Light weight transformer model based on ViT	✗	✗	Moderate	Low	Excellent for classification and transfer learning
PSPEdgeWeedNet	✓	✓	High	Moderate	Improved boundary localization, efficient deep feature extraction, better fine-grained segmentation

## Ablation study: model and improvements

### Impact of class weights and boundary-aware loss on PSPNet model performance

The Table 5 presents experimental results with varying class weights for crop, weed, and background to determine the most optimal weight configuration for each class. The evaluation is based on both training and validation metrics. Upon analyzing the results, it is evident that the class weights of 2 for crop, 3 for weed, and 1 for background yield the highest accuracy compared to all other configurations tested. This combination consistently demonstrated superior performance in both training and validation accuracy, making it the most effective choice for achieving optimal model performance in this context.

**Table 5:** Baseline PSPNet with different class weights. The significant values are highlighted in bold.

Class weights	Training loss	Validation loss	Training accuracy	Validation accuracy
*Crop* = 2*,Weed* = 5*, Background* = 1	0.2777	0.3959	0.898	0.8897
*Crop* = 3*,Weed* = 7*, Background* = 1	0.2804	0.4073	0.8832	0.8763
*Crop* = 6*,Weed* = 15*, Background* = 1	0.2696	0.4619	0.8417	0.8455
*Crop* = 2*,Weed* = 3*, Background* = 1	**0.2482**	**0.3755**	**0.9098**	**0.8960**

The ablation results shown in Table 6 clearly demonstrate the incremental improvements achieved by incorporating boundary-aware loss and edge detection branch into the base PSPNet architecture:

- Baseline Improvement with Boundary-Aware Loss: Adding boundary-aware loss alone improves precision, F1 score, and IoU (from baseline values) and moderately enhances overall segmentation quality, achieving an F1 score of 0.7068 and IoU of 0.5875. This suggests it helps the model better capture class boundaries, reducing misclassifications.
- Impact of Edge Detection Branch: Replacing boundary loss with an edge detection branch significantly boosts all metrics, particularly IoU (0.68) and F1 score (0.7897). This indicates that edge cues contribute more effectively to object separation and delineation than boundary loss alone.
- Combined Strategy- Proposed Model: The full integration of both edge detection and boundary-aware loss in the proposed PSPEdgeWeedNet model leads to the best performance across all metrics.

**Table 6:** Ablation study showing the impact of boundary-aware loss and edge detection branch on PSPNet-based segmentation model performance (Significant values are highlighted in bold).

Model variant	Accuracy	Precision	Recall	F1 Score	IoU
PSPNet + Class weights + Boundary Aware Loss	0.8943	0.6945	0.7236	0.7068	0.5875
PSPNet + Class weights + Edge Detection Branch	0.9279	0.7800	0.8003	0.7897	0.6800
PSPNet + Class weights + Boundary Aware Loss + Edge Detection Branch (Proposed model)	**0.9335**	**0.9399**	**0.9335**	**0.9358**	**0.8797**

**Fig. 13:**
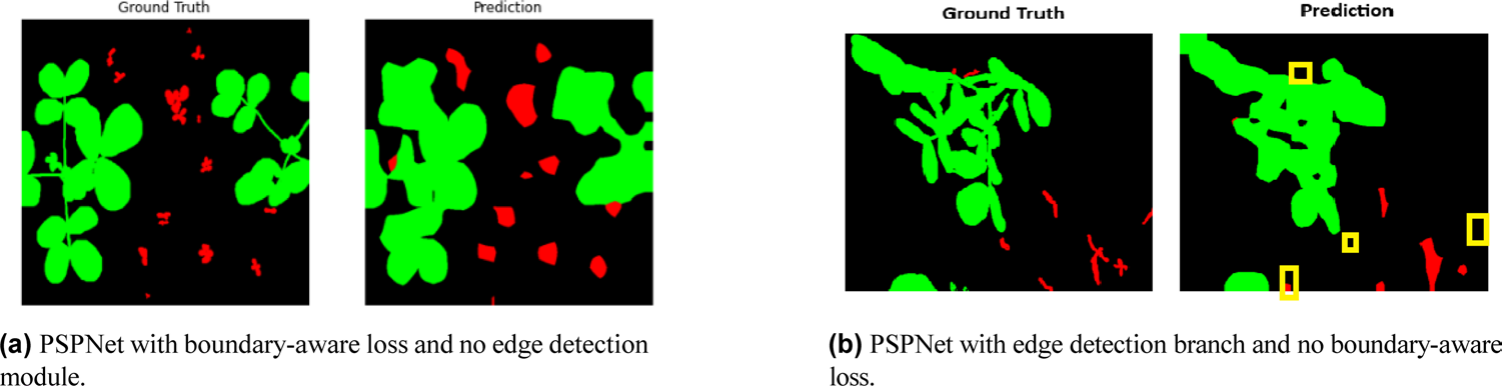
Visualisation of ground truth and predictions for two PSPNet variations. Green color represents crop, red color for weed, and black color for the background.

This significant performance jump highlights the complementary effect of both strategies in enhancing segmentation quality, particularly in challenging cases involving complex boundaries and overlapping vegetation. Fig 13 presents the visualization of ground truth and predictions for two models: (a) PSPNet with boundary-aware loss but without the edge detection module, and (b) PSPNet with the edge detection module but without boundary-aware loss. While both models effectively capture the crop regions, instances of weed misclassification as crops and missed weed detections are observed in both cases.

### Impact of different backbone architectures on PSPEdgeWeedNet performance

The performance comparison of PSPEdgeWeedNet with three different backbone networks, ResNet101, ResNet18, and MobileNetv2 are shown in the Table 7. Results demonstrates that the ResNet101 backbone yields the best results across all

**Table 7:** Performance of PSPEdgeWeedNet with different backbone architectures (Significant values are highlighted in bold).

Model	Precision	Recall	F1 Score	IoU	Accuracy
PSPEdgeWeedNet (ResNet101)	**0.9399**	**0.9335**	**0.9358**	**0.8797**	**0.9335**
PSPEdgeWeedNet (ResNet18)	0.7580	0.8111	0.7825	0.6444	0.9194
PSPEdgeWeedNet (MobileNetv2)	0.8015	0.8074	0.8050	0.6730	0.9320

key evaluation metrics. Specifically, it achieves a precision of 0.9399, recall of 0.9335, F1-score of 0.9358, IoU of 0.8797, and accuracy of 0.9335. In contrast, the ResNet18 version shows lower scores (F1-score: 0.7825, IoU: 0.6444), while the MobileNetV2 variant performs slightly better than ResNet18 (F1-score: 0.805, IoU: 0.673), although still below ResNet101. The superior performance of PSPEdgeWeedNet with ResNet101 confirms that deeper and more expressive backbones significantly improve segmentation quality in agricultural applications. However, lighter backbones like ResNet18 or MobileNetV2 may still be suitable where inference speed or resource constraints are critical, at the cost of some accuracy. Fig 14 shows the visualization of ground truth and predictions generated by PSPEdgeWeedNet using ResNet18 and MobileNetv2 as backbone networks. When ResNet18 is used as the backbone, all crop instances are accurately identified, and a few weed instances are detected. However, some weeds are misclassified as crops. In contrast, with MobileNetV2 as the backbone, the model fails to detect weed instances, as highlighted by the yellow boxes in the visualization.

**Fig. 14:**
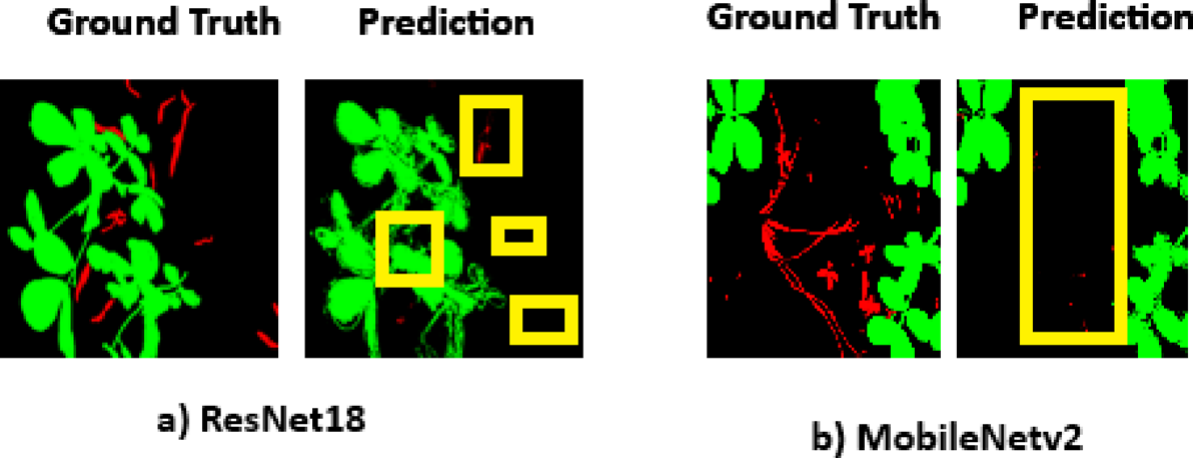
Visualization of ground truth and predictions generated using PSPEdgeWeedNet with ResNet18 and MobileNetv2 as backbone networks. Green color represents crop, red color for weed, and black color for the background. Yellow boxes highlight misclassifications and missed detections.

### Impact of data augmentation on PSPEdgeWeedNet model performance

A comprehensive set of data augmentation techniques was used on the proposed PSPEdgeWeedNet model. Color-based transformations were applied using ColorJitter, which randomly altered image brightness, contrast, saturation, and hue to mimic the diverse lighting conditions commonly encountered in agricultural environments. Geometric augmentations included horizontal flipping with a 50% probability and vertical flipping with a 30% probability, helping the model learn orientation- invariant features. Additionally, random rotations up to 15 degrees were incorporated to accommodate angular variations during image capture. To further diversify spatial configurations, a RandomAffine transformation was applied, introducing minor translations, scaling, and shearing. Lastly, Gaussian blur was used to simulate image quality degradation such as motion blur or defocus, encouraging the model to learn more robust visual patterns.

From the results obtained as shown in Table 8, it is clear that data augmentation did not improve the model’s performance. It led to a noticeable decrease in accuracy, precision, recall, and F1 score. The drop in performance suggests that the augmented data might not have been beneficial in this specific scenario, potentially due to overfitting or a misalignment between the augmented data and the actual data distribution. Therefore, for the PSPEdgeWeedNet model, using no data augmentation seems to lead to better overall performance. On both the training and validation sets, PSPEdgeWeedNet (without data augmentation) performs better than PSPEdgeWeedNet (with data augmentation) in every parameter, including accuracy, precision, recall, and F1 score.

**Table 8:** Ablation experiments comparing PSPEdgeWeedNet performance with and without data augmentation. Significant values are highlighted in bold.

Metric	PSPEdgeWeedNet (Without Data Augmentation)	PSPEdgeWeedNet (With Data Augmentation)
Accuracy	**0.9335**	0.8293
Precision	**0.9399**	0.8241
Recall	**0.9335**	0.8293
F1 Score	**0.9358**	0.8263

**Fig. 15:**
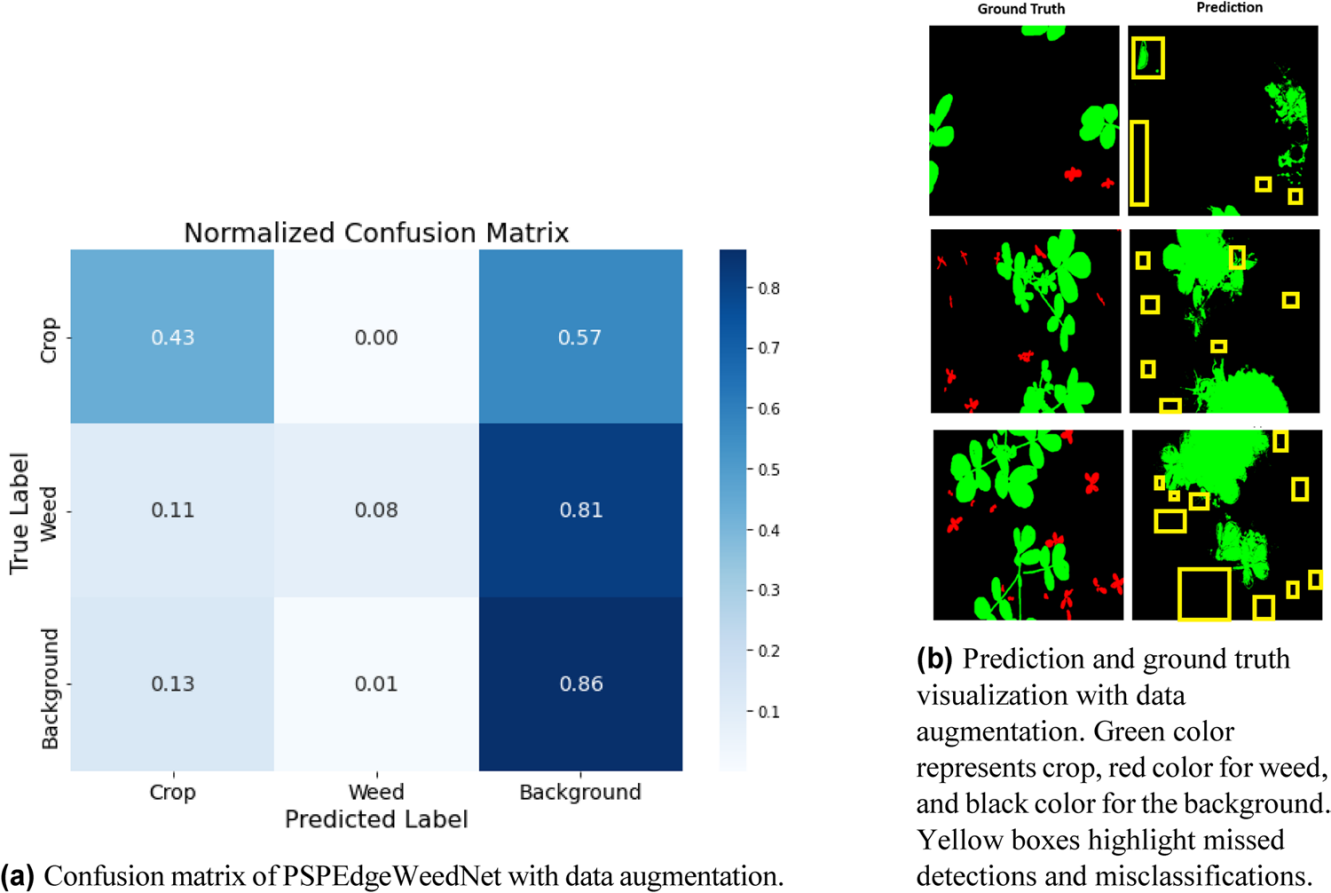
Evaluation results of PSPEdgeWeedNet with data augmentation.

Figure. 15a displays the confusion matrix generated for PSPedgeWeedNet with data augmentation. Fig 15b illustrates the visualization of ground truth and prediction for the same. Based on the results presented in the above table, as well as insights drawn from the confusion matrix and the visual comparisons of predictions with ground truth, it is evident that applying data augmentation had a detrimental impact on the model’s performance. Specifically, data augmentation not only led to a decline in key evaluation metrics such as accuracy, precision, recall, and F1-score, but also contributed to an increase in both misclassifications and missed detections. A significant number of weed instances were left undetected in the augmented dataset, indicating that the augmentation techniques may have distorted critical features required for effective weed identification.

Furthermore, the predictions refined using Conditional Random Fields (CRF) revealed that the boundaries between crop and weed regions were not sharply or accurately delineated when data augmentation was applied. This lack of clear segmentation negatively affected the model’s ability to distinguish between classes at the object level.

In contrast, our proposed model, PSPEdgeWeedNet without data augmentation, consistently produced more accurate and well-defined predictions. It not only achieved superior quantitative performance but also demonstrated better qualitative outcomes in terms of segmentation clarity and object boundary definition. Hence, it can be concluded that the non-augmented training pipeline yields more reliable results for crop–weed segmentation tasks in this specific dataset.

## Analysis of findings

The performance of PSPNet enhanced with boundary-aware loss and no edge detection module revealed inherent limitations in accurately delineating the boundaries between crop and weed classes. Specifically, the model failed to capture sharp edge definitions, resulting in blurred contours and reduced structural fidelity—particularly in the morphology of weed leaves. This led to imprecise segmentation and instances of class confusion, where crop regions were incorrectly classified as weeds, highlighting difficulties in achieving effective class separation.

In contrast, PSPEdgeWeedNet exhibited notable improvements in boundary localization compared to the baseline PSPNet. Misclassifications were substantially reduced, and edge contours were more distinguishable. However, the segmentation output still lacked the precision required for fine-grained structural representation, especially in the detailed contours of weed foliage, which remained partially undefined and unstructured.

The integration of Conditional Random Fields (CRF) in the visualization module of PSPEdgeWeedNet significantly enhanced the boundary refinement process. This led to more continuous and precise delineation of crop and weed edges, with improved preservation of the morphological and structural characteristics of vegetation. Visual inspection of the outputs confirmed this enhancement, although the model still struggled with detecting extremely small weed instances, which were occasionally omitted during segmentation.

A qualitative visualization comparison was also conducted between UNet, SegNet, DeepLabv3, Swin-Unet, lightweight transformer based on ViT, and the proposed PSPEdgeWeedNet. In the case of UNet, although the model generally succeeded in identifying both crops and weeds, several instances of weed misclassification as crops were observed, highlighted using yellow bounding boxes. SegNet exhibited a higher degree of misclassification than UNet, with weeds frequently mislabeled as crops and, in some cases, background pixels erroneously detected as weeds. Swin-Unet demonstrated relatively high segmentation accuracy; however, visualizations revealed inaccuracies along the boundaries of crops and weeds, with occasional misclassifications where crops were incorrectly segmented as weeds.

In contrast, PSPEdgeWeedNet produced more refined and accurate boundary segmentation than all the aforementioned models. The contours of both crop and weed regions were sharply defined, and no significant misclassifications were observed in the visual outputs. Nevertheless, a minor limitation persisted—extremely small weed patches were sometimes not detected. Based on both quantitative evaluation (in terms of precision, recall, F1-score, and IoU) and qualitative visual inspection, it can be concluded that PSPEdgeWeedNet outperforms other segmentation architectures. It delivers superior boundary delineation, significantly reduced misclassification, and enhanced structural preservation of vegetation classes, making it highly effective for semantic segmentation tasks in agricultural applications.

## Future work

The PSPEdgeWeedNet model, which segments peanut field images into crop, weed, and background classes, has shown effectiveness in segmenting images. However, there are several areas for future exploration. Firstly, diversifying the dataset to include images from a wider range of crops which can improve the model’s generalizability across different agricultural scenarios. Secondly, expanding the dataset to include a wider variety and an increased number of weed species will enhance the model’s robustness and enable accurate differentiation between diverse weed types. Thirdly, by combining the PSPEdgeWeedNet model with UAVs, ground robots, or smartphone applications, it can be enhanced to detect weeds in actual agricultural settings. To make the model more scalable and appropriate for long-term deployment in dynamic agricultural ecosystems, further research could employ adaptive learning approaches to change with new data over time.

Importantly, the current architecture is optimized for RGB-only inputs, with a focus on real-time deployment in agricultural field conditions. Future work will address the incorporation of spectral diversity (e.g., multispectral and NIR data) and include multi-crop validation to ensure broader applicability and improved performance in varied agricultural systems.

## Conclusion

This paper presents PSPEdgeWeedNet, a novel semantic segmentation architecture specifically developed for precise crop and weed identification in agricultural field conditions. The proposed model builds upon the foundational Pyramid Scene Parsing Network (PSPNet) by integrating an edge-aware module that enhances the model’s capability to delineate object boundaries with higher fidelity. This architectural augmentation enables the extraction of both global contextual information and localized edge features, which is particularly critical for distinguishing between visually similar plant structures such as weeds and crops, especially in heterogeneous field environments where occlusion, overlapping foliage, and background clutter are prevalent.

Quantitative and qualitative evaluations demonstrate that PSPEdgeWeedNet surpasses the UNet, SegNet, PSPNet, DeepLabv3, Swin-Unet, and lightweight transformer model based on ViT in both segmentation performance and boundary localization. PSPEdgeWeedNet shows significant improvements in edge sharpness, reduced crop-to-weed misclassification, and improved retention of structural features such as weed leaf morphology. Although Conditional Random Fields (CRF) are not incorporated into the training or inference stages, their application in post-processing for visualization highlights the enhanced boundary preservation achieved by PSPEdgeWeedNet. The visual results suggest that the model is capable of generating continuous, well-preserved boundaries and exhibits reduced fragmentation, independent of any CRF-based post-processing.

Furthermore, PSPEdgeWeedNet demonstrates improved sensitivity to small and sparsely distributed weed instances, al- though some extremely tiny weed structures remain difficult to accurately segment. Despite achieving high overall segmentation accuracy, certain challenges persist, notably suboptimal Intersection over Union (IoU) scores for underrepresented classes. These results confirm the efficacy of the proposed edge-aware enhancements in addressing core limitations of conventional semantic segmentation models and suggest potential avenues for future work in class-imbalance mitigation and fine-grained structure recovery in agricultural vision tasks.

## Data Availability

The datasets presented in this study can be found in online repositories(https://github.com/ptdkhoa/Peanut-dataset).
